# Inside the Envelope: Endogenous Retrovirus-K Env as a Biomarker and Therapeutic Target

**DOI:** 10.3389/fmicb.2015.01244

**Published:** 2015-11-13

**Authors:** Marie-Josée Nadeau, Mamneet Manghera, Renée N. Douville

**Affiliations:** ^1^Douville Lab, Department of Biology, University of WinnipegWinnipeg, MB, Canada; ^2^Department of Immunology, University of ManitobaWinnipeg, MB, Canada

**Keywords:** endogenous retrovirus, envelope protein, cancer, immune response, therapeutics

## Abstract

Due to multiple ancestral human retroviral germ cell infections, the modern human genome is strewn with relics of these infections, termed endogenous retroviruses (ERVs). ERV expression has been silenced due to negative selective pressures and genetic phenomena such as mutations and epigenetic silencing. Nonetheless, select ERVs have retained the capacity to be damaging to their host when reawakened. Much of the current research on the ERVK Env protein strongly suggests a causal or contributive role in the pathogenesis of various cancers, autoimmune and infectious diseases. Additionally, there is a small body of research suggesting that ERVK Env has been domesticated for use in placental development, akin to the ERVW syncytin. Though much is left to ascertain, the innate immune response to ERVK Env expression has been partially characterized and appears to be due to a region located in the transmembrane domain of the Env protein. In this review, we aim to highlight ERVK Env as a biomarker for inflammatory conditions and explore its use as a future therapeutic target for cancers, HIV infection and neurological disease.

## Introduction

Over evolutionary time, endogenous retroviruses (ERVs) have integrated into the human genome by germ-cell infections of human ancestors. Infection of germ cells would ensure that all the cells of the progeny of the infected individual would contain and continue to transmit the ERV sequence (Hohn et al., 2013). In healthy humans, select ERVs are expressed homeostatically in a tissue-specific fashion without any negative consequences to the host. Thus, it would appear that overly pathogenic strains have been lost to negative selective pressures (Voisset et al., 2008). Likewise, pathogenicity is further dampened as many ERVs have been silenced due to the accumulation of deleterious mutations and deletions, and epigenetic phenomena such as methylation (Voisset et al., 2008). In the genome of modern humans, ~8% of genetic information can be attributed to ERVs.

The endogenous retrovirus-K (ERVK) species, especially the HML-2 clade, is one of the most recently endogenized human retroviruses and retains open reading frames (ORFs) capable of encoding functional proteins, making it the most intact and biologically active ERV group to date (Hohn et al., 2013). In certain disease states, select ERVK loci can be reactivated, and overall upregulation of ERVK expression has been associated with several cancers, inflammatory, infectious, and autoimmune diseases. However, it remains unclear how ERVK is implicated in the progression of disease—whether activation of the provirus plays a causal or contributive role in pathogenesis, or if its reactivation is a bystander consequence of inflammation or hormone dysregulation in disease states (Golan et al., 2008; Manghera and Douville, 2013; Reis et al., 2013).

## ERVK envelope protein

**Figure 1:**
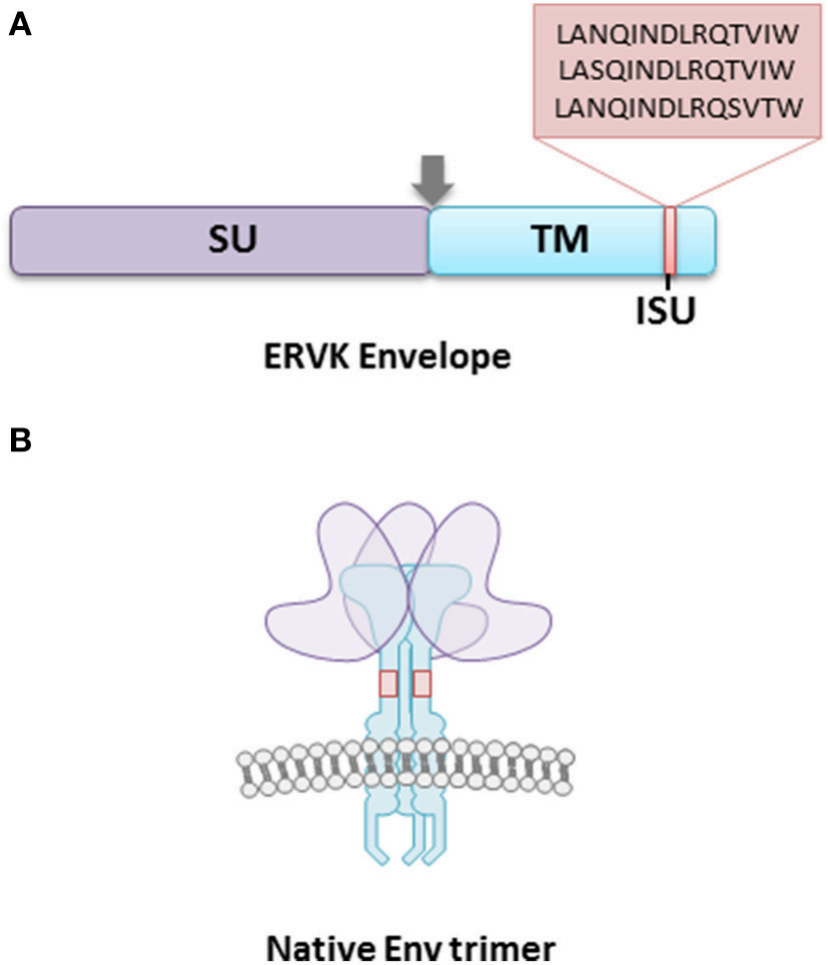
**The ERVK envelope protein is composed of surface (SU) and transmembrane (TM) subunits**. **(A)** The TM subunit contains an immunosuppressive (ISU) domain, which is postulated to contribute toward host immune-modulation. The majority of ERVK sequences contain the ISU domain sequence LANQINDLRQTVIW, with a minority having the sequences LASQINDLRQTVIW, and LANQINDLRQSVTW. The arrowhead represents a consensus furin cleavage site. **(B)** The surface ERVK Env protein is a trimer of SU and TM heterodimers. The TM anchors the viral receptor into the host cell membrane, and is essential in the fusion of virion and host cell membranes. The SU provides host cell receptor specificity, although the cellular receptors for ERVK Env are unknown.

Accumulating evidence points toward the role of ERVK envelope (Env) protein in health and disease. Based on the research that is available on ERVK Env protein, it is known to exert neurotoxic and immunosuppressive effects (Morozov et al., 2013; Li et al., 2015), although the mechanisms underlying these events are incompletely understood. The ERVK Env is composed of a 55-kDa surface (SU) unit, which is responsible for receptor binding, and a 39-kDa fusogenic transmembrane (TM) unit (Figure 1).

It appears that a specific region within the ectodomain of the TM subunit, referred to as the immunosuppressive (ISU) domain, may be responsible for ERVK immunomodulation, or at least contribute greatly to it. The TM protein of ERVK is similar to that of many other retroviruses (although not all retroviral TM proteins contain an ISU domain), as it tends to remain conserved (Henzy and Johnson, 2013; Morozov et al., 2013). The ISU is located in the N-terminal end of the ectodomain, adjacent to the conserved cysteine residues. The vast majority of ERVK sequences contain the ISU domain sequence LANQINDLRQTVIW, with a minority having the sequences LASQINDLRQTVIW, and LANQINDLRQSVTW (Morozov et al., 2013). Interestingly, it was found that when the HIV-1 TM subunit gp41 was selectively mutated in the ISU domain, the virus was not able to effectively infect host cells (Morozov et al., 2012). Thus, the ISU domain is crucial for host cell infectivity and modulating cytokine production in infected cells.

A recent study performed using ERVK virus-like particles, a recombinant ERVK TM and a peptide corresponding to the conserved region of the ERVK TM, revealed that the immune response elicited against the protein appears to share many parallels with that targeting HIV-1 TM gp41(Morozov et al., 2013). It was found that the proteins were able to modulate production of numerous cytokines, inhibit the activation of immune cells, and induce changes in the transcription levels of hundreds of genes (Morozov et al., 2013). In one of the assays performed, it was found that the ERVK TM was able to inhibit the activation of PBMCs in both human and murine cells in a dose-dependent fashion (Morozov et al., 2013). Furthermore, the ERVK TM was found to induce the overexpression of the following cytokines: IL-6, IL-8, IL-10, MCP-1, RANTES, MIP-1α, MIP-1β, uPAR, sTNFRII, and GCSF (Morozov et al., 2013). In particular, the immunosuppressive IL-10 was expressed in significantly higher amounts (Morozov et al., 2013). When analysing changes in gene expression, over 300 genes were upregulated in response to the TM protein and over 300 were downregulated (Morozov et al., 2013). Among the 10 most upregulated genes were (in order of highest to lowest fold change) MMP-1, IL-6, IL-1A, CXCL13, CCL7, TREM1, CXCL1, ARNT2, CA12, and KIAA1295 (Morozov et al., 2013). The nine most downregulated genes (in order of highest to lowest fold change) were SEPP1, FCN1, DHRS9, FCN2, HS3ST2, TREM2, ALDH1A1, GPR34, and KCNJ35 (Morozov et al., 2013). The results of this study are consistent with previous data suggesting the ERVK TM is immunosuppressive. As well, it is important to note that the recombinant TM protein and the peptide corresponding to the ISU domain of the TM elicited similar immune responses; this suggests that this domain is likely responsible for the bulk of the protein's immunomodulatory activity (Morozov et al., 2013).

Moreover, another study has demonstrated that ERVK Env is able to antagonize the activity of tetherin, an innate immune protein which functions in preventing the budding of enveloped viruses from an infected cell (Lemaître et al., 2014). Consequently, ERVK re-activation can prove to be detrimental for resolution of infections with exogenous enveloped viruses, such as HIV-1. Lastly, research has also suggested that the ERVK TM can supress T cell activation via its modulation of dendritic cells (Hummel et al., 2015).

## ERVK Env in health and disease states

### Placental development

Though the majority of the research on ERVK focuses on its contributions to disease, other research suggests that it may play a role in normal physiology. It has been suggested that the TM subunit of ERVK envelope protein may be implicated in placentogenesis and pregnancy (Kammerer et al., 2011). The ERVK Env has been found to be expressed in villous and extravillous cytotrophoblast cells, both of which are components of the placenta (Kammerer et al., 2011). As this protein is known to have immunosuppressive properties, it is thought that it may play a role in protecting the fetus from the maternal immune system. Due to its expression pattern in placental tissue and fusogenic properties, it is believed that the ERVK TM may play a role in cell-cell fusion, similar to the envelope proteins ERVW syncytin and ERV-FRD syncytin 2. The functional redundancy of these ERV Env proteins may ensure placentogenesis in the face of variations in ERV expression (Kammerer et al., 2011).

### ERVK Env in cancers

Currently, the implications of enhanced ERVK Env expression in the pathogenesis of human cancers has yet to be fully elucidated (Downey et al., 2014). However, even with the limited knowledge that is available in this area of ERVK research, several novel cancer treatments have focused on exploiting the expression of ERVK Env on the surface of tumor cells.

Research has shown that ERVK tends to be overexpressed in cancers of the reproductive system, lymphoid organs, myeloid organs, breast, prostate, and urinary bladder (Singh, 2007; Wallace et al., 2014; Wang-Johanning et al., 2014). As a biomarker, ERVK Env or antibodies targeting ERVK Env may be independent disease indicators or paired with current disease markers to improve diagnostic and prognostic reliability (Wallace et al., 2014; Wang-Johanning et al., 2014). It has been proposed that this protein may contribute to carcinogenesis by triggering cell-cell fusion and possibly encouraging tumor proliferation and metastasis (Hohn et al., 2013). Moreover, as ERVK Env is known to have immunosuppressive properties, it may putatively provide protection to tumors by helping them evade immune surveillance, similar to that of the fetal-maternal interface (Kammerer et al., 2011; Hohn et al., 2013).

It is interesting to note that some melanoma cell lines (SKMel-28, SKMel-1, 518A2, MelJuso, HS-Mel2 and JH-Mel6, and V-Mel7) were able to be produce virus-like particles which contained mature ERVK Gag and Env proteins (Hohn et al., 2013; Downey et al., 2014). Additionally, it has been reported by several groups that T47D human mammary carcinoma cell lines have been able to produce virus-like particles containing ERVK-related sequences (Seifarth et al., 1998; Contreras-Galindo et al., 2015). Although the retroviral-like particles produced demonstrated reverse transcriptase activity and were able to enter neighboring cells, they proved to be unable to undergo integration into the host genome (Contreras-Galindo et al., 2015).

#### Breast cancer

**Figure 2:**
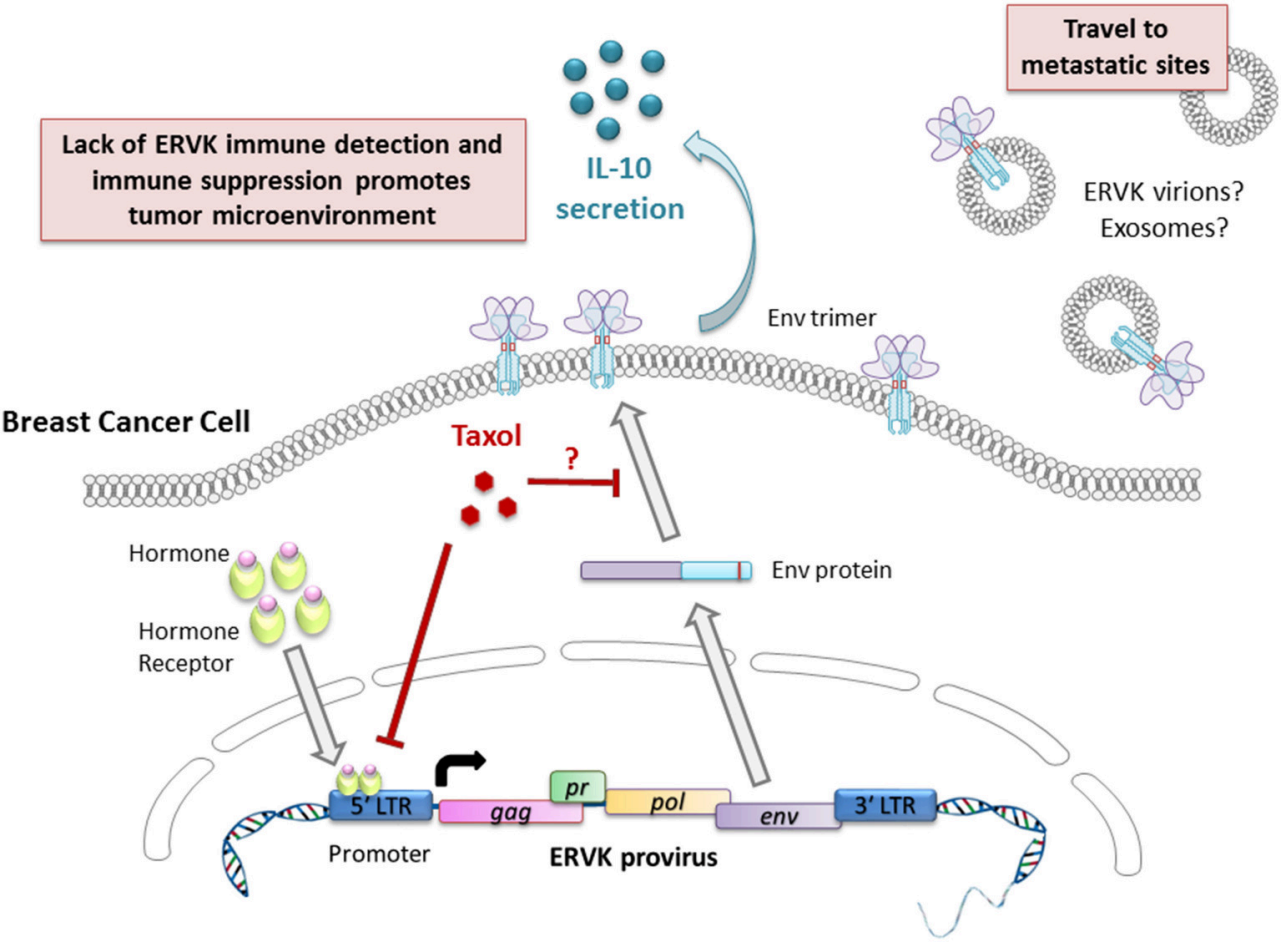
**Putative involvement of ERVK Env in breast cancer pathology and progression**. The ERVK viral promoter is responsive to androgens, estrogens, progestogens, and glucocorticoid hormones, thus favoring viral transcription and the production of Env protein. Taxol limits ERVK *env* expression and may prevent ERVK Env trafficking to the cell surface by modulating microtubule assembly. Cell surface expression of Env promotes secretion of the cytokine IL-10, which is a potent modulator of immune responses. Secretion of retroviral virions or exosomes carrying retroviral cargo may promote cellular transformation at metastatic sites.

The reactivation and increased expression of ERVK Env has been linked to the majority of malignant breast tumors (Wang-Johanning et al., 2012; Cegolon et al., 2013; Downey et al., 2014; Figure 2). In studies involving American and Chinese breast cancer patients, it was found that the ERVK Env protein by itself was an indicator of poor prognosis and lymph node metastasis (Zhao et al., 2011). Furthermore, another study also determined that ERVK *env* transcripts in blood plasma or serum were significantly lower in breast cancer patients undergoing treatment, in contrast to primary breast cancer patients. As well, patients being treated with taxotere or taxol had the lowest levels of *env* transcripts (Rhyu et al., 2014), although the mechanism of *env* suppression remains to be elucidated.

The innate and adaptive immune responses to ERVK Env in breast tumors have been partially characterized. ERVK Env has been demonstrated to elicit both B and T cell responses in breast cancer patients (Wang-Johanning et al., 2008). Notably, significant titers of anti-ERVK Env IgG antibodies where detected in the majority of breast cancer patients (Wang-Johanning et al., 2008, 2012). *In vitro*, PBMC from breast cancer patients that were stimulated with autologous dendritic cells pulsed with ERVK SU antigens activated T cell responses against ERVK, which resulted in the proliferation of T cells, the production of IFN-γ, and cytokine secretion (Wang-Johanning et al., 2008). When cytokine secretion in response to the ERVK antigens was analyzed, it was observed that a T-helper 1 cytokine dominated response was elicited, as characterized by measurement of IL-2, IL-6, IL-8, and IL-10 (Wang-Johanning et al., 2008). As well, it was found that ERVK-specific cytotoxic T lymphocytes were capable of killing cells expressing ERVK Env in breast cancer patients (Wang-Johanning et al., 2008).

Nonetheless, the presence of the ERVK Env in breast cancer tumors does need to be viewed as a bad omen. Though this protein is a marker of malignant tumors, the results of recent studies have given hope that this protein may be targeted in novel breast cancer therapies. In xenograft mice studies, it was found that anti-ERVK Env monoclonal antibodies were able to inhibit the growth of and induce apoptosis in tumor cells (Wang-Johanning et al., 2012; Cegolon et al., 2013). Though additional research is needed in this area, exploiting ERVK-specific expression in breast cancer tumors is a promising avenue for the development of novel targeted immunotherapies.

#### Melanoma

Within the *env* gene of ERVK (HML-6) lies an ORF for a pseudo-gene known as ERVK-MEL (Schiavetti et al., 2002; Katoh et al., 2011; Cegolon et al., 2013). The protein product of this ORF is an antigenic peptide that has been found to be significantly expressed in the majority of benign tumors, such as normal and dysplastic naevi, as well as in malignant tumors such as sarcomas, lymphomas, and bladder and breast cancers (Schiavetti et al., 2002; Cegolon et al., 2013). ERVK-MEL is considered to be a marker of increased risk of melanoma and has been found to be expressed in 85% of malignant melanocytes (Schiavetti et al., 2002). In addition, cytotoxic T cells have been shown to recognize this antigen and mount an adaptive immune response (Schiavetti et al., 2002). Moreover, it is interesting to add that there exists a sequence homology between ERVK-MEL and the epitopes in the Bacillus of Calmette Guerin and vaccina virus vaccine, as well as the yellow fever virus vaccine (Krone et al., 2005). This has led some to believe that these vaccines may be able to play a preventative role in melanoma development due to the cross-reactivity of the adaptive immune response against ERVK-MEL (Krone et al., 2005).

The expression of the HML-2 Env protein has also been associated with melanomas (Büscher et al., 2006). The ERVK Env has been detected in primary and metastatic melanoma biopsies and melanoma cell lines, but not in melanocytes or lymph nodes (Büscher et al., 2005). Unfortunately, there is evidence suggesting that the antibody response to the ERVK Env protein in early to mid-stage melanoma is negatively correlated with patients' chances of survival (Hahn et al., 2008). Little is known about the role of this protein in melanoma or the innate immune response it elicits.

ERVK envelope protein remains a candidate target for novel melanoma treatments by the fact that it is a tumor-associated antigen can be exploited (Krishnamurthy et al., 2015). In a mouse xenograft study, it was found that T cells genetically engineered to target the ERVK Env protein were able to exert significant anti-tumor effects on metastasized melanoma tumors expressing the ERVK Env in an antigen-specific fashion (Krishnamurthy et al., 2015). Although further work is required to translate this treatment for human use, it remains a promising therapeutic option for advanced stage melanoma (Krishnamurthy et al., 2015).

#### Lymphoma

It is thought that ERVK18 superantigen (Sag) may play a role in the development of some lymphomas (Sutkowski et al., 2004; Gross et al., 2011). Non-specific activation of T cells by ERVK18 SAg may enhance cytokine secretion, although its exact role in inflammation and tumorigenesis has yet to be confirmed or debunked. Epstein Barr virus (EBV) has been associated with the development of lymphoma, and is known to transactivate the expression of ERVK18 Env (Sutkowski et al., 2004). Thus, EBV-triggered ERVK18 SAg expression may contribute to lymphoma by triggering the expansion of self-reactive T cells via stimulating Vβ_7_ T cells and the subsequent breakdown of host immunity (Stauffer et al., 2001). As the ERVK18 SAg is also known to be activated by the antiviral cytokine IFNα, an inflammatory response following other exogenous virus infections may be sufficient to enhance ERVK18 Sag expression (Stauffer et al., 2001). It is possible that the superantigen activity of ERVK18 Env may play a role in human carcinogenesis analogous to that of mouse mammary tumor virus (MMTV) in murine carcinomas. Expression of viral SAg ensures the establishment of a viral reservoir of infected and proliferating lymphocytes, and delivery of virus to lymph nodes and the mammary gland (Ross, 2010). Moreover, it is worth mentioning that the levels of ERVK RNA viral load in the blood of lymphoma patients have been observed to drop following therapy (Contreras-Galindo et al., 2008). This finding points to the possibility of utilizing ERVK titers as a tool in monitoring therapeutic progress.

### ERVK Env in autoimmune and infectious disease

Though much of the research on ERVK Env and disease pertains to its association to tumorigenesis, this viral protein is also thought to be implicated in several autoimmune disorders, such as insulin-dependent diabetes mellitus (IDDM), multiple sclerosis (MS), and rheumatoid arthritis (RA) (Sicat et al., 2005; Dickerson et al., 2008; de la Hera et al., 2013; Mason et al., 2014). Current data suggests a link between ERVK and autoimmunity, however, the details of mechanisms by which it may occur remain to be fully elucidated. ERVK has also been shown to be reactivated and implicated in HIV infection (reviewed in van der Kuyl, 2012). Interestingly, ERVK Env expression in the brain is postulated to be neuroprotective in HIV infection (Bhat et al., 2014). In contrast, it has recently been shown that enhanced expression of ERVK envelope protein contributes to neuronal DNA damage and motor neuron death in a transgenic mouse model (Li et al., 2015), highlighting that ERVK can drive neuropathogenic outcomes.

## Modulation of the immune response by ERVK Env

Though the mechanism by which the ERVK envelope glycoprotein triggers an immune response and contributes to immune suppression continues to evade us, general knowledge of the immune response to ERVK has been partially characterized. One prominent feature of the immune response to ERVK Env, in particular the TM subunit, is a spike in the production of IL-10 (Morozov et al., 2013). IL-10 is an immunosuppressive cytokine that impedes the expression of inflammatory cytokines, MHC class II antigens and costimulatory molecules on macrophages, as well as promotes B cell proliferation and antibody production (Couper et al., 2008). This is consistent with the observation that the ERVK envelope protein is frequently expressed at the surface of tumor cells which appears to allow them to remain undetected by the immune system and still allow the recognition of ERVK Env as a non-self target, as evidenced by an ERVK Env-specific antibody response (Hahn et al., 2008; Wang-Johanning et al., 2012; Reis et al., 2013). This mechanism may account for the immune protection of the fetus during pregnancy, as ERVK Env has been found to be expressed in placental tissues along with elevated levels of IL-10 (Kammerer et al., 2011; Morozov et al., 2013). Considering that NF-κB signaling mediates the induction of ERVK (Manghera and Douville, 2013; Manghera et al., 2015), the counter-mechanism of ERVK Env to induce IL-10 and hinder the inflammatory response and NF-κB activity may represent a viral immune evasion strategy. NF-κB is known to be involved in the JAK-STAT signaling pathway, whose deregulation is implicated in various cancers (Boudny and Kovarik, 2002). As ERVK is found to be transcriptionally activated in many cancers, it is possible that ERVK Env induction of anti-inflammatory cytokines protects both cancerous tumors and ERVK from immune detection. Thus, we hypothesize that having its envelope glycoprotein expressed at the cell surface represents a strategy by which the ERVK provirus can protect the tumor environment in which it is up-regulated.

Despite immunomodulation driven by IL-10, there is evidence that the adaptive immune system can detect and respond to ERVK Env antigen. Specifically, anti-ERVK Env antibodies can facilitate antibody-dependent cytotoxicity in HIV infected cells (Michaud et al., 2014). In a complementary manner, ERVK Env-derived peptides can also be potent T cell epitopes (Garrison et al., 2007; SenGupta et al., 2011; Jones et al., 2012). CD8^+^ T cells responding to the peptide sequence CIDSTFNWQHR within ERVK Env, were able to kill cells expressing their cognate peptide (Jones et al., 2012).

As well, several studies have demonstrated that despite being unable to produce productive infections, ERVK virus-like particles (VLPs) are able to enter cells (Contreras-Galindo et al., 2015). This means it is possible that the envelope protein may be detected upon viral entry by either cell surface TLR4 or other innate immune sensors (Altfeld and Gale, 2015; Duperray et al., 2015; Hurst and Magiorkinis, 2015). It also alludes to the possibility that VLPs or exosomes carrying viral protein cargo may mediate tumorigenesis at metastatic sites (Balaj et al., 2011; Lokossou et al., 2014).

## Conclusions

Despite the limited knowledge that is currently available on the ERVK envelope protein, it has great potential for use in diagnosing, monitoring, and treating human illness. The utility of ERVK Env as a blood biomarker stems from its reliability in discriminating between individuals with cancer and without. However its use independently of co-indicators may be limited due to enhanced expression not only in cancers, but also in infectious and neurological diseases (Garrison et al., 2007; Li et al., 2015). In combination with suitable biomarkers of a given condition, detection of ERVK Env itself or anti-ERVK Env antibody titers can enhance diagnostic and prognostic readouts (Wallace et al., 2014; Wang-Johanning et al., 2014).

As this protein is a tumor-associated antigen, it is possible to use it to develop targeted therapies that could result in fewer side effects, due to the specificity of anti-ERVK Env antibodies. This could lead to not only more successful treatment, but a higher quality of life for those undergoing treatment. Several independent groups of researchers have shown that an anti-ERVK Env vaccine is a promising cancer treatment as it has been effective in targeting several types of malignant tumors via the inhibition of tumor growth and induction of apoptosis (Downey et al., 2014). Moreover, there exists the possibility of conjugating cytotoxic drugs to ERVK Env-specific antibodies to increase the effectiveness and specificity of treatment (Wang-Johanning et al., 2012; Downey et al., 2014). Additionally, modulating cytotoxic killing of cells expressing ERVK Env antigens (Jones et al., 2012; Michaud et al., 2014), through interferon treatment, expansion of relevant T cell subsets or small molecules promoting effector mechanisms, may prove useful in eradicating malignant cells. Finally, as the upregulation of ERVK transcripts is well-documented in many disease states, and its downregulation during or following treatment, it is possible to use this as a marker of therapeutic progress in numerous types of cancers.

More resources need to be allocated toward gaining a deeper understanding of ERVK biology, its Env protein and the role it plays in disease. This would allow for us to not only further exploit the ERVK envelope as a molecular target for novel immunotherapies, but to also gain new insights into the nature of the pathophysiology of various diseases with which the activated provirus is associated. This emphasizes the need for an ERVK Proteome Map, as it would lend itself as a valuable tool in recognizing and identifying ERV proteins as potential therapeutic targets.

## Author contributions

Wrote the paper: MN, MM, and RND. Designed the artwork for the figures: MM and RND.

## Funding

This work was sponsored by the Dr. Beni Sahai Fund for Cellular and Molecular Biology for Advancement in Medical Research (2014–2015), in support of undergraduate research at the University of Winnipeg.

### Conflict of interest statement

The authors declare that the research was conducted in the absence of any commercial or financial relationships that could be construed as a potential conflict of interest.
